# Supplementary material for: Early macrophage response to obesity encompasses Interferon Regulatory Factor 5 regulated mitochondrial architecture remodelling
Source: Nat Commun. 2022 Aug 30;13:5089. doi: 10.1038/s41467-022-32813-z (PMC9427774; doi:10.1038/s41467-022-32813-z)

Early macrophage response to obesity encompasses Interferon Regulatory Factor 5 regulated mitochondrial architecture remodelling

Orliaguet et al.

Supplementary information

Item	Page
1. Supplementary Figures	
a. Supplementary Figure 1	2
b. Supplementary Figure 2	4
c. Supplementary Figure 3	6
d. Supplementary Figure 4	8
e. Supplementary Figure 5	10
f. Supplementary Figure 6	12
g. Supplementary Figure 7	14
h. Supplementary Figure 8	16
i. Supplementary Figure 9	18
2. Supplementary Tables	
a. Supplementary Table S1	20
b. Supplementary Table S2	20
c. Supplementary Table S3	21
3. Supplementary Methods	
a. Gating strategies	22

Supplementary Figures

Supplementary Figure 1

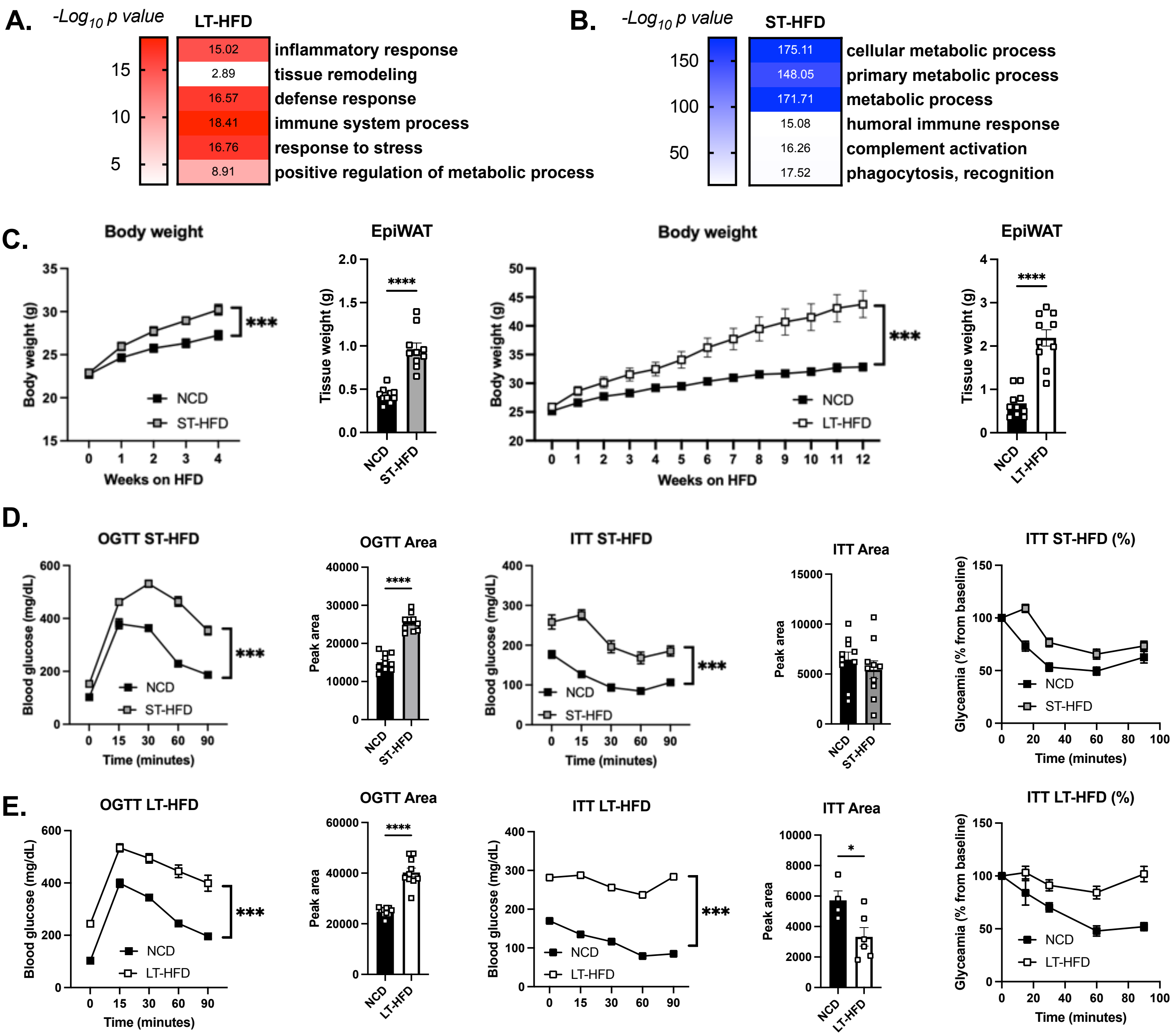

Figure S1. IRF5-transcriptome is associated with altered cellular metabolic adaptation and mice on short-term high-fat diet lose glycaemic homeostasis over time

A. & B. WT and IRF5-KO mice were placed on a long-term (LT-) high-fat diet (HFD) (**A**) or a short-term (ST-HFD) (**B**). F4/80^{+} adipose tissue macrophages (ATMs) were sorted from epididymal fat pads (EpiWAT) for RNA sequencing (RNA-seq). Differentially expressed genes (-log10 p > 1.3) between genotypes were used for gene ontology (GO) term enrichment. Heatmaps represent p values (n=4 per genotype).

C. Body weight and EpiWAT weight of C57BL/6J mice on NCD, ST-HFD (left) or LT-HFD (right) (n=10 per group, ANOVA for weight curves and two-tailed unpaired t-test for tissue weight, left-to-right, ****p=0.0000018 and ****p=0.0000012).

D. & **E.** Oral glucose tolerance test (OGTT) and insulin tolerance test (ITT) performed on mice following NCD, ST-HFD (**D.**) or LT-HFD (**E.**), and peak area of the corresponding curves (n=10 for NCD and ST-HFD, n=4 for NCD and n=6 for LT-HFD; ANOVA for curves and two-tailed unpaired t-test for area, left-to-right, ****p=0.0000000049 (**D.**), ****p=0.000000088 and *p=0.029).

Data are presented as mean ± SEM. Source data are provided as a Source Data file

Supplementary Figure 2

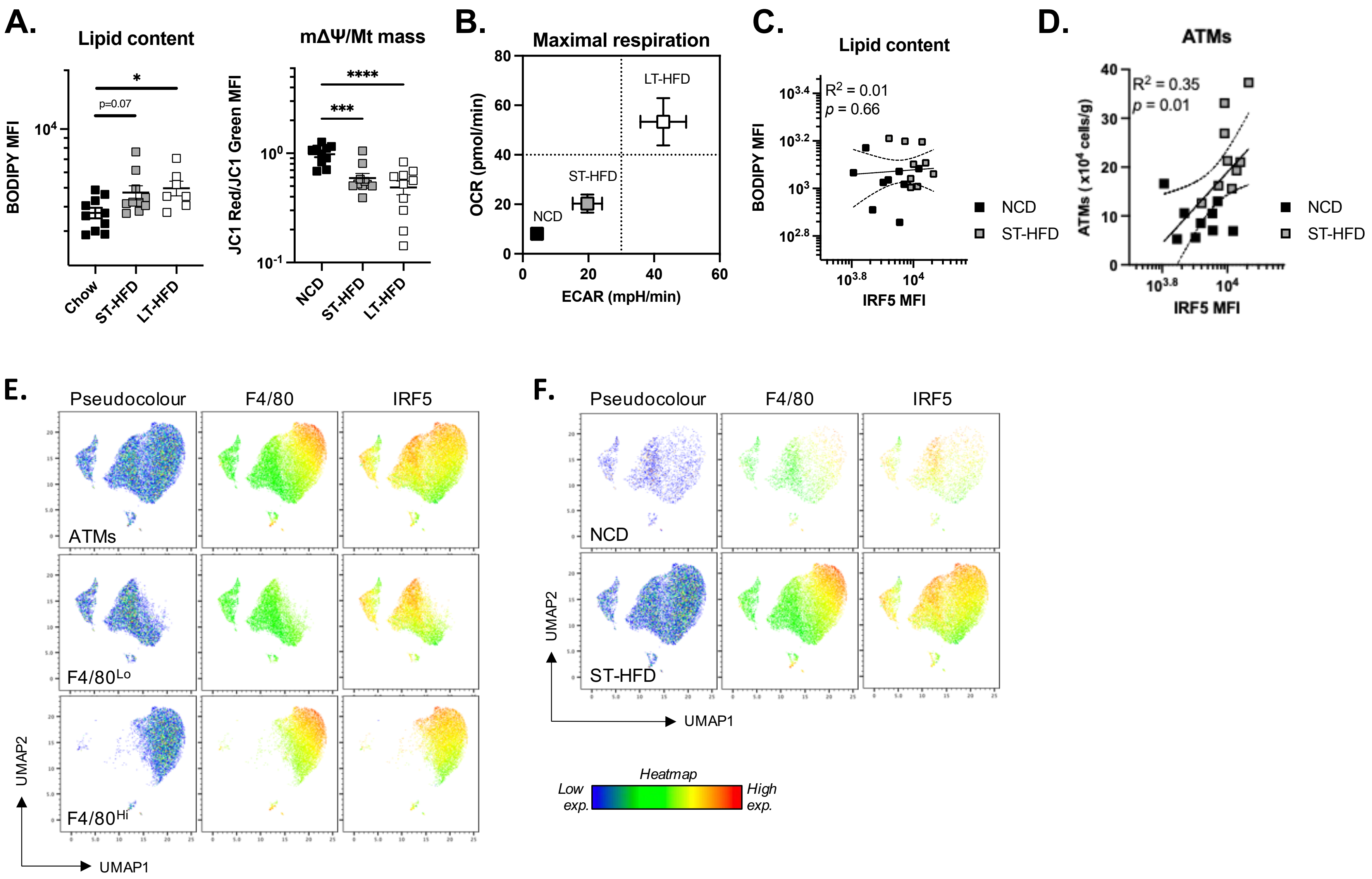

Figure S2. Adipose tissue macrophages undergo metabolic adaptation on high-fat diet and IRF5 is highly expressed in $F4/80^{Hi}$ cells

A. Median fluorescence intensity (MFI) of BODIPY and JC1-Red/JC1-Green ratio to assess lipid content and mΔΨ/Mt mass respectively, in epididymal white adipose tissue (EpiWAT) $F4/80^{+}$ $CD11b^{+}$ macrophages (ATMs) of C57BL/6J mice on a normal chow diet (NCD), short-term (ST-) high-fat diet (HFD) or a long-term (LT-)HFD (n=10 per group in NCD and ST-HFD; n= 7 in LT-HFD; One-way ANOVA **p*=0.042, multiple comparisons **p*=0.043, ****p*=0.00065 *****p*=0.000029).

B. Energetic plot with extracellular acidification rate (ECAR) and oxygen consumption rate (OCR) from maximal respiration of extracellular flux analysis (Fig. 1E.). Assay performed on $F4/80^{+}$ magnetically sorted from the EpiWAT of C57BL/6J mice on NCD (n=4), ST-HFD (n=9) or LT-HFD (n=8).

C. Correlative analysis between IRF5 and BODIPY MFI (Linear regression, Pearson correlation, $R^2 = 0.01$; two-tailed $p = 0.66$) in EpiWAT $F4/80^{+}$ $CD11b^{+}$ ATMs of C57BL/6J mice on NCD or ST-HFD (n=9 per group).

D. Correlative analysis between IRF5 MFI in EpiWAT $F4/80^{+}$ $CD11b^{+}$ ATMs and EpiWAT ATM content (Linear regression, Pearson correlation, $R^2 = 0.35$; two-tailed $p = 0.0104$) of C57BL/6J mice on NCD or ST-HFD (n=9 per group).

E. UMAP projection of IRF5 expression across ATMs, $F4/80^{Lo}$ and $F4/80^{Hi}$ cells from the EpiWAT of mice upon NCD or ST-HFD.

F. UMAP projection of IRF5 expression in ATMs across on NCD or ST-HFD.

Data are presented as mean ± SEM. Source data are provided as a Source Data file.

Supplementary Figure 3

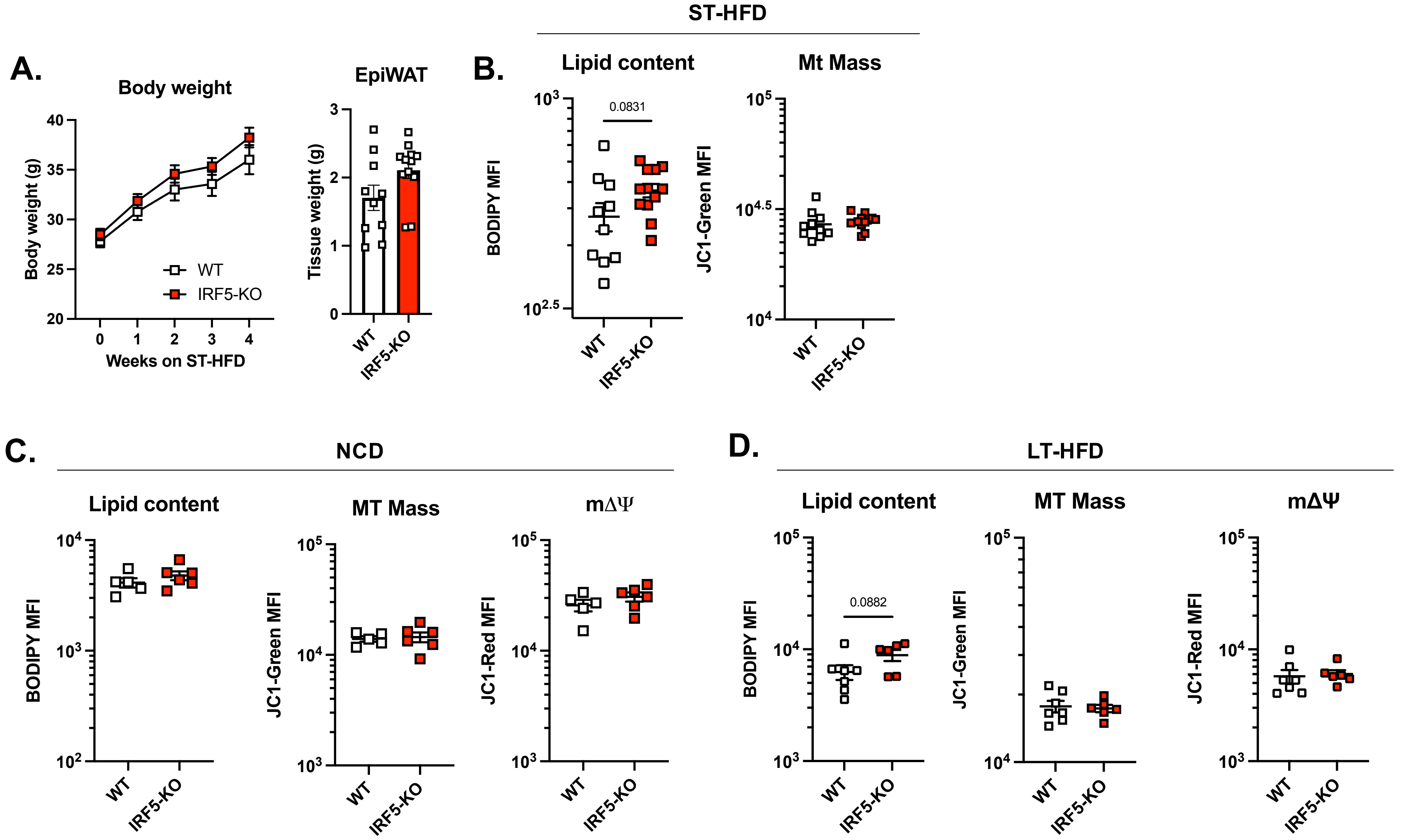

Figure S3. Normal chow and long-term high-fat diet are not associated with adaptive cellular metabolism in IRF5-deficiency.

A. Body weight and epididymal white adipose tissue (EpiWAT) weight of WT and IRF5-KO mice on short-term (ST-) high-fat diet (HFD) (n=10 for WT and n=12 for IRF5-KO)

B. MFI of BODIPY and JC1-Green in EpiWAT F4/80^{+} CD11b^{+} macrophages of WT and IRF5-KO mice on ST-HFD (n=10 for WT and 12 for IRF5-KO, two-tailed unpaired t-test, *p*=0.0831).

C. & D. MFI of BODIPY, JC1-Green and JC1-Red in EpiWAT F4/80^{Hi} macrophages of WT and IRF5-KO mice on normal chow diet (NCD) (**C**) or long term (LT-) HFD (**D**) (n=5 WT and 6 IRF5-KO mice on NCD, n=7 WT and n=6 IRF5-KO on LT-HFD, two-tailed unpaired t-test, *p*=0.0882).

Data are presented as mean ± SEM. Source data are provided as a Source Data file.

Supplementary Figure 4

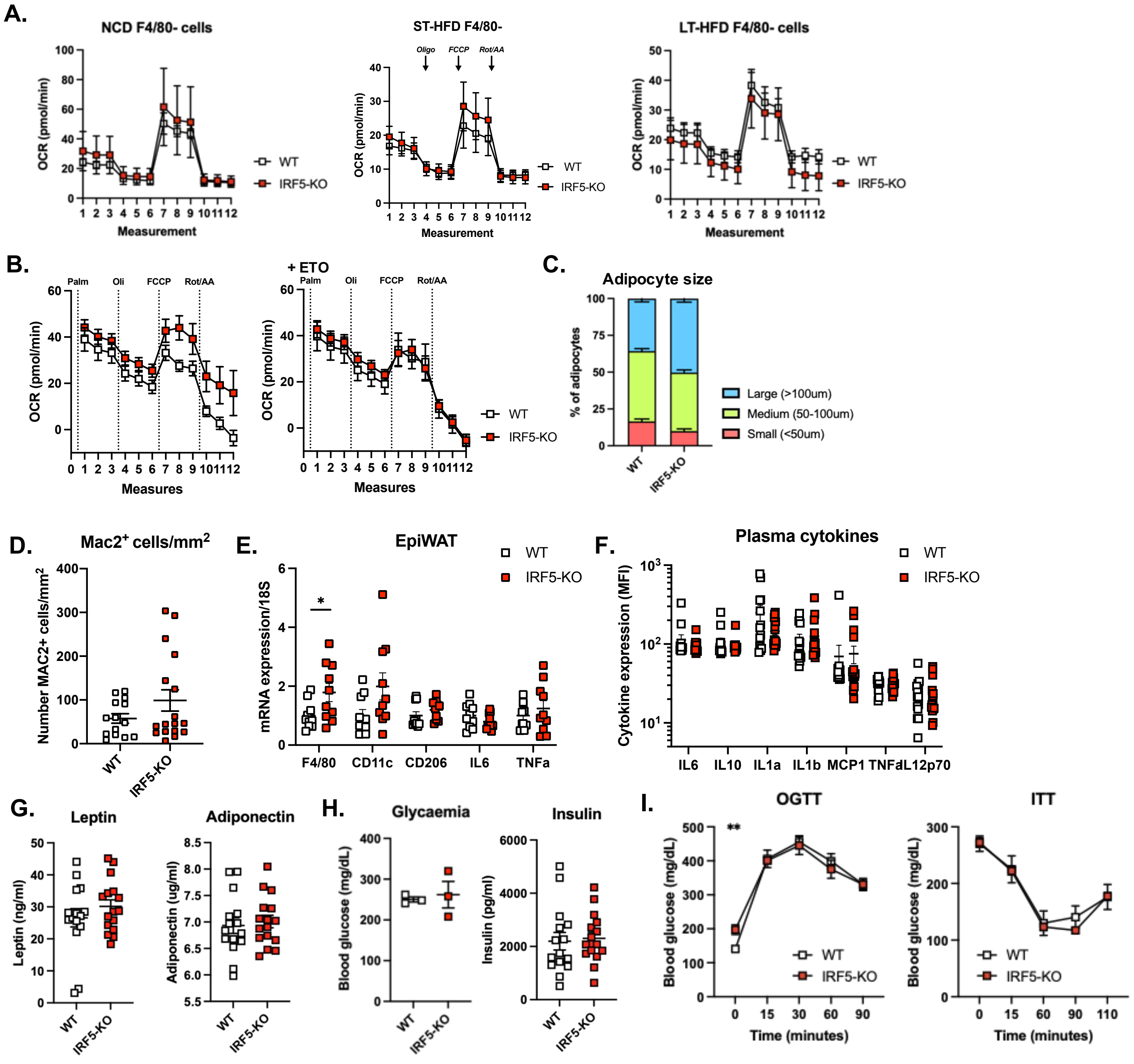

Figure S4. F4/80- cells and inflammatory status of adipose tissue are not affected by IRF5-deficiency upon short-term high-fat diet

A. Oxygen consumption rate (OCR) from extracellular flux analysis with oligomycin (Oli), carbonyl cyanide 4-(trifluoromethoxy) phenylhydrazone (FCCP) and Rotenone/Antimycin A (Rot/AA) administration. Analysis on F4/80- cells of the stromal vascular fraction (SVF) of epididymal white adipose tissue (EpiWAT) from WT and IRF5-KO mice on a normal chow diet (NCD; left), short-term (ST-) high-fat diet (HFD; middle) and long-term (LT-) HFD (right) (n=4 mice per genotype for NCD, n=6 WT and 9 IRF5-KO mice for ST-HFD and n=6 WT and 5 IRF5-KO mice for LT-HFD).

B. OCR from extracellular flux analysis, following palmitate with or without etomoxir, Oligo, FCCP and Rot/AA were administered. Cells were from SVF of EpiWAT of WT and IRF5-KO mice on ST-HFD (n=7 WT and 9 IRF5-KO mice).

C. Proportions of small (<50 um), medium (50-100 um) or large (>100 um) adipocytes in EpiWAT of WT and IRF5-KO mice on ST-HFD (Related to Fig 2E).

D. Number of Mac2+ cells on EpiWAT sections of WT and IRF5-KO mice on ST-HFD (n=14 WT and 17 IRF5-KO mice) (Related to Fig 2E, F).

E. F4/80, CD11c, CD206, IL6 and TNFa mRNA expression normalized to 18S, in EpiWAT of WT and IRF5-KO mice on ST-HFD (n=10 mice per genotype, two-tailed unpaired t-test **p*= 0.027)

F. Plasma cytokine quantification from WT and IRF5-KO mice on ST-HFD (n=14 WT and n=16 IRF5-KO mice). Median fluorescence intensity (MFI) of IL6, IL10, IL1a, IL1b, MCP1, TNFa or IL12p70 coated beads.

G. Plasma levels of leptin and adiponectin from WT and IRF5-KO mice on ST-HFD (n=15 WT and n=16 IRF5-KO mice).

H. Glycaemia (n=3 per genotype) and plasma insulin levels (n=15 WT and n=16 IRF5-KO mice) from WT and IRF5-KO mice on ST-HFD.

I. Oral glucose tolerance test (OGTT) and insulin tolerance test (ITT) performed on WT and IRF5-KO mice following ST-HFD (n=8 for WT and n=5 for IRF5-KO).

Data are presented as mean ± SEM. Source data are provided as a Source Data file.

Supplementary Figure 5

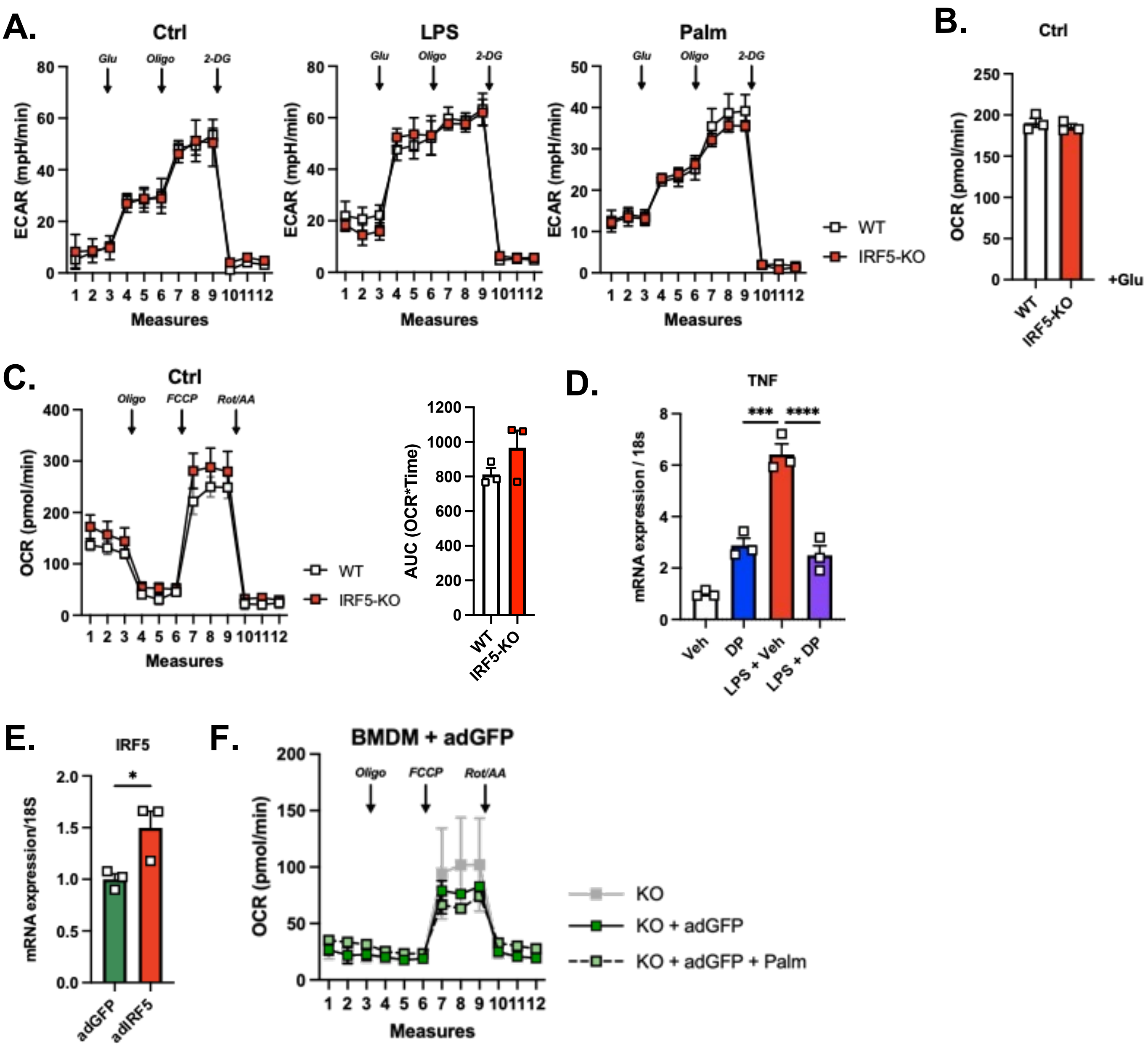

Figure S5. IRF5-deficiency does not alter mitochondrial respiration under basal conditions nor glycolysis under basal and treated conditions

A. Bone marrow-derived macrophages (BMDM) from WT and IRF5-KO mice were treated with lipopolysaccharides (LPS) or palmitate (Palm) for 24 hours. Extracellular acidification rate (ECAR) was measured in extracellular flow analysis following addition of glucose (Glu), oligomycin (Oligo) and 2-deoxyglucose (2-DG) (n=5 per genotype for treated conditions and n=3 per genotype for control).

B. Oxygen consumption rate (OCR) following glucose administration in extracellular flux analysis of untreated WT and IRF5-KO BMDMs, in Fig S5A (n=5 per genotype).

C. OCR measurements during extracellular flux analysis, with Oligo, carbonyl cyanide 4-(trifluoromethoxy) phenylhydrazone (FCCP) and Rotenone/Antimycin A (Rot/AA) administration (left) and area under the curve (right) of untreated WT and IRF5-KO BMDMs (n=3 per genotype).

D. BMDMs from C57BL/6J mice were treated with an IRF5-decoy peptide (DP) or a vehicle (Veh) and with LPS. RNA expression of TNF was quantified, and normalized to 18S (n=3 per genotype and per condition, ***p=0.0002 and ****p<0.0001, one-way ANOVA).

E. BMDMs from IRF5-KO mice were treated with an IRF5 adenovirus (adIRF5) or a control adenovirus (adGFP). IRF5 RNA expression was quantified (n=3 per group; *p=0.04, two-tailed unpaired t-test).

F. Metabolic flux analysis of BMDMs from IRF5-KO mice were treated with an adGFP and with Palm (n=3 per group). Cells were treated with Oligo, FCCP and Rot/AA.

Data are presented as mean ± SEM. Source data are provided as a Source Data file.

Supplementary Figure 6

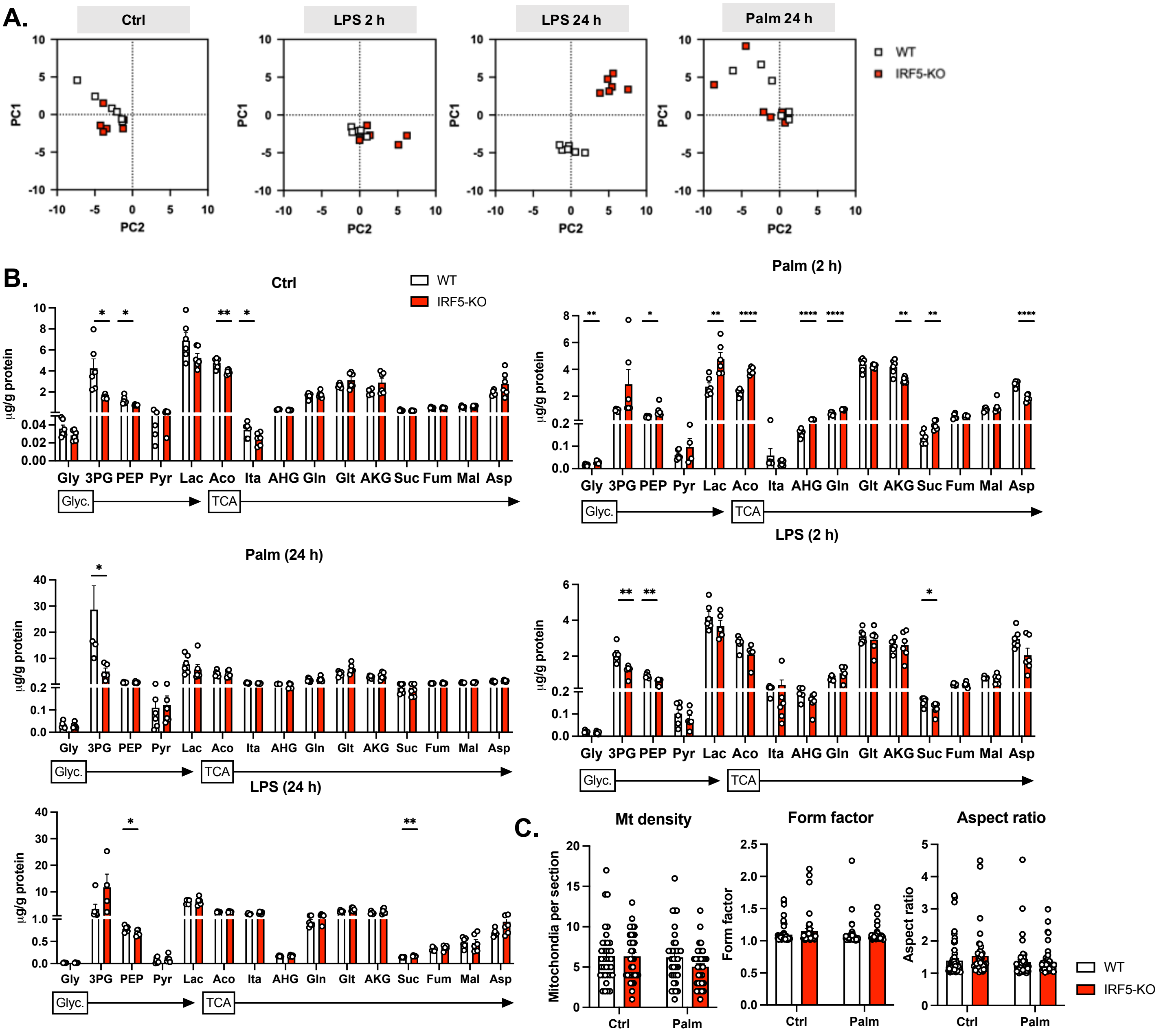

Figure S6. TCA metabolite quantification and mitochondrial dynamics in bone marrow-derived macrophages from IRF5-KO and WT mice following treatment with LPS and palmitate

A. Principal component analysis (PCA) on TCA cycle metabolites in WT and IRF5-KO bone marrow-derived macrophages (BMDMs) left untreated, or stimulated with bacterial lipopolysaccharides (LPS) for 2 or 24 h or with palmitate (Palm) for 24 h.

B. Absolute quantification of TCA metabolites in BMDMs from WT and IRF5-KO mice, left untreated, stimulated with LPS or Palm for 2 or 24 h (n=6 per genotype and per condition; unpaired t-test, left to right, Ctrl: *p=0.013, *p=0.013, **p=0.002, *p=0.04; LPS 2h: **p=0.0041, **p=0.0068, *p=0.038; LPS 24h: *p=0.02 **p=0.002; Palm 2h: **p=0.001, *p=0.03, **p=0.004, ****p<0.0001, ****p=0.000005, ****p=0.00006, **p=0.002, **p=0.002 ****p=0.000015; Palm 24h: *p= 0.02).

C. Analysis of mitochondrial density, form factor and aspect ratio from electron micrographs in WT and IRF5-KO BMDMs treated with Palm for 2 h or in untreated controls (Ctrl). Cells derived from n=3 independent animals per genotype, 5 different whole-cell images (Mt Density) and 10 different zoom images (form factor, aspect ratio) per sample and per condition were analyzed (unpaired t-test)

Data are presented as mean ± SEM. Source data are provided as a Source Data file.

Supplementary Figure 7

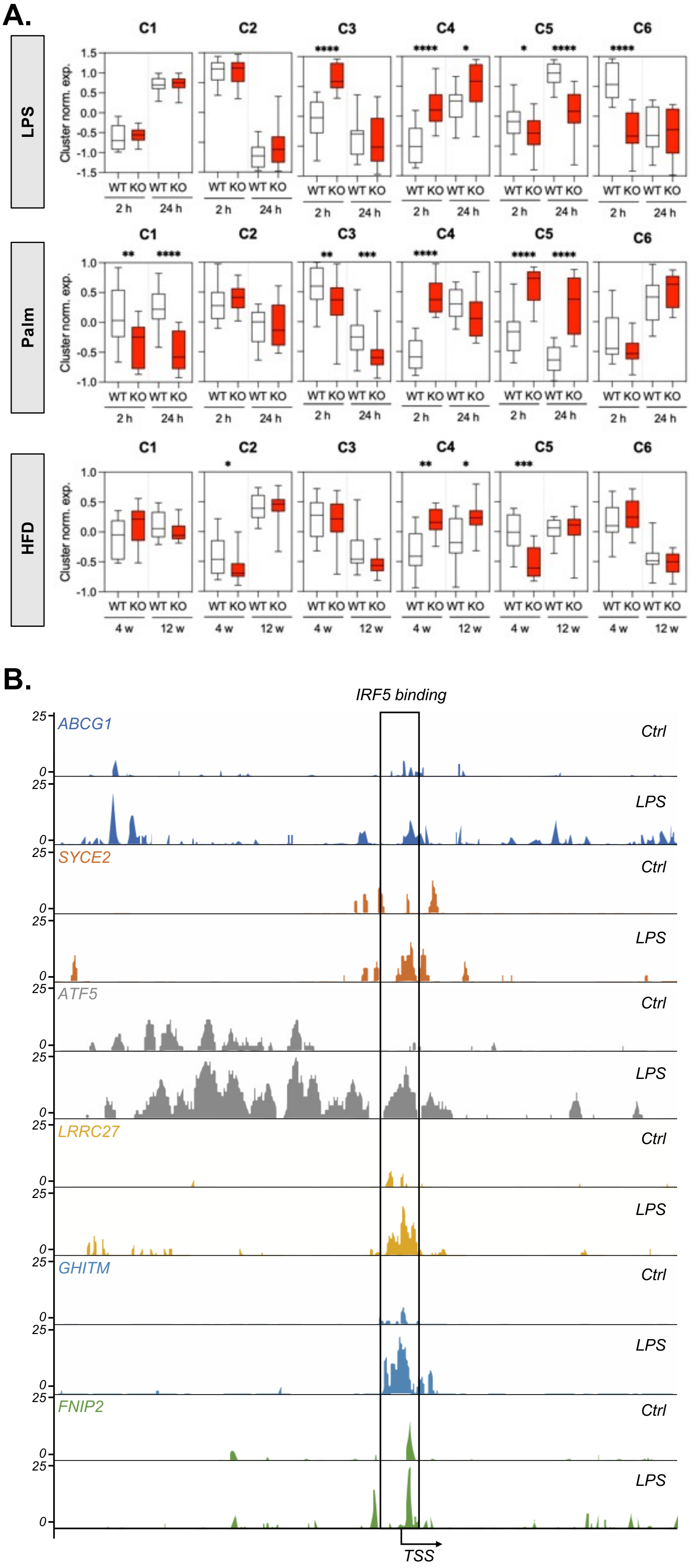

Figure S7. IRF5-cistrome and transcriptomic analysis in IRF5-KO and WT bone-marrow derived and adipose tissue macrophages upon stimulation.

A. Normalized mRNA expression per cluster identified in Fig 5A (left to right: LPS ****p<0.0001, ****p<0.0001, *p=0.028, *p=0.031, ****p<0.0001, ****p<0.0001; Palm **p=0.016, ****p<0.0001, **p=0.0038, ***p=0.0003, ****p<0.0001, ****p<0.0001; HFD *p=0.013, **p=0.0017, *p=0.0156, ***p=0.0001; one-way ANOVA). n=3 per condition and per genotype for Ctrl, LPS or Palm treated cells, n=4 per time-point and per genotype for HFD-fed animals. Box-and-whiskers plots represent: line at median, boxes cover 25th and 75th percentiles and whiskers (error bars) extend to minimum and maximum values. Ctrl: control; LPS: lipopolysaccharides; Palm: Palmitate; HFD: High-fat diet.

B. Gene track from ChIP-seq in Fig 5C. of overlapping genes in all conditions with IRF5 binding, in control (Ctrl) condition or upon lipopolysaccharide (LPS) stimulation. n=2 biological replicates analysed for differential binding, pooled for visualization.

Data are presented as mean ± SEM. Source data are provided as a Source Data file.

Supplementary Figure 8

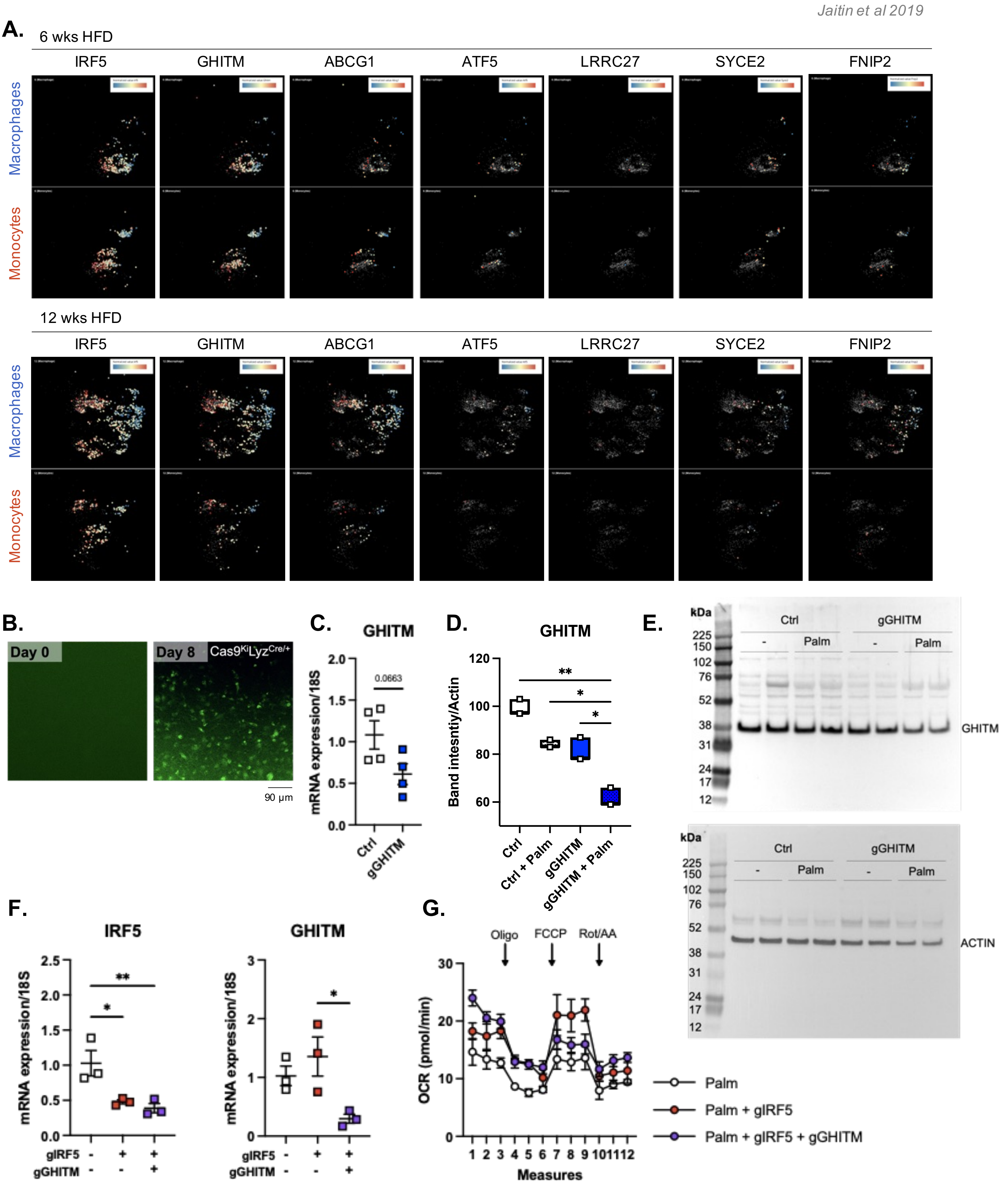

Figure S8: GHITM is highly expressed in epididymal visceral white adipose tissue macrophages and monocytes relative to other IRF5 targets.

A. Public dataset of single-cell RNA sequencing of the epididymal white adipose tissue stromal vascular fraction of C57BL/6J mice following 6 or 12 weeks of high-fat feeding. Macrophages and monocytes were identified and expression of IRF5, of GHITM and of ABCG1, SYCE2, FNIP2, ATF5 and LRRC27 were projected onto tSNE plots per cell type and duration of high-fat feeding.

B. Fluorescent image of bone marrow-derived macrophages (BMDMs) from mice with myeloid-restricted Cas9-GFP at day 0 and day 8 of differentiation (20 x magnification, scale bar indicated 90 μm). Representative image of 2 independent experiments where each experiment included n=3 biologically independent samples.

C. Gene expression of GHITM in Cas9-expressing BMDMs treated with lipofection agent (Ctrl) or with a gRNA targeting GHITM (gGHITM) (n=4 per condition, two-tailed unpaired t-test p=0.0663).

D. Quantification of western blotting against GHITM in the same experiment as Fig. 6E (n=2 per condition, one-way ANOVA. **p=0.0046, *p=0.031, *p=0.043).

E. Full membranes of Fig. 6E western blotting.

F. IRF5 and GHITM expression (normalized to 18S), in Cas9-expressing BMDMs following transfection with a gRNA targeting IRF5 (gIRF5), double transfection with gGHITM and gIRF5 or with lipofection agent alone (Ctrl) (n=3 per condition, one-way ANOVA, *p=0.022, **p=0.009 for IRF5; and *p=0.019 for GHITM).

G. Oxygen consumption rate and maximal respiration from extracellular flux analysis on Palm-treated BMDMs following transfection with a gRNA targeting IRF5 (gIRF5), double transfection with gGHITM and gIRF5 or with lipofection agent alone (Ctrl). Cells were treated with oligomycin (Oli), carbonyl cyanide 4-(trifluoromethoxy) phenylhydrazone (FCCP) and Rotenone/Antimycin A (Rot/AA) administration (n=3 per condition).

Data are presented as mean ± SEM. Source data are provided as a Source Data file.

Supplementary Figure 9

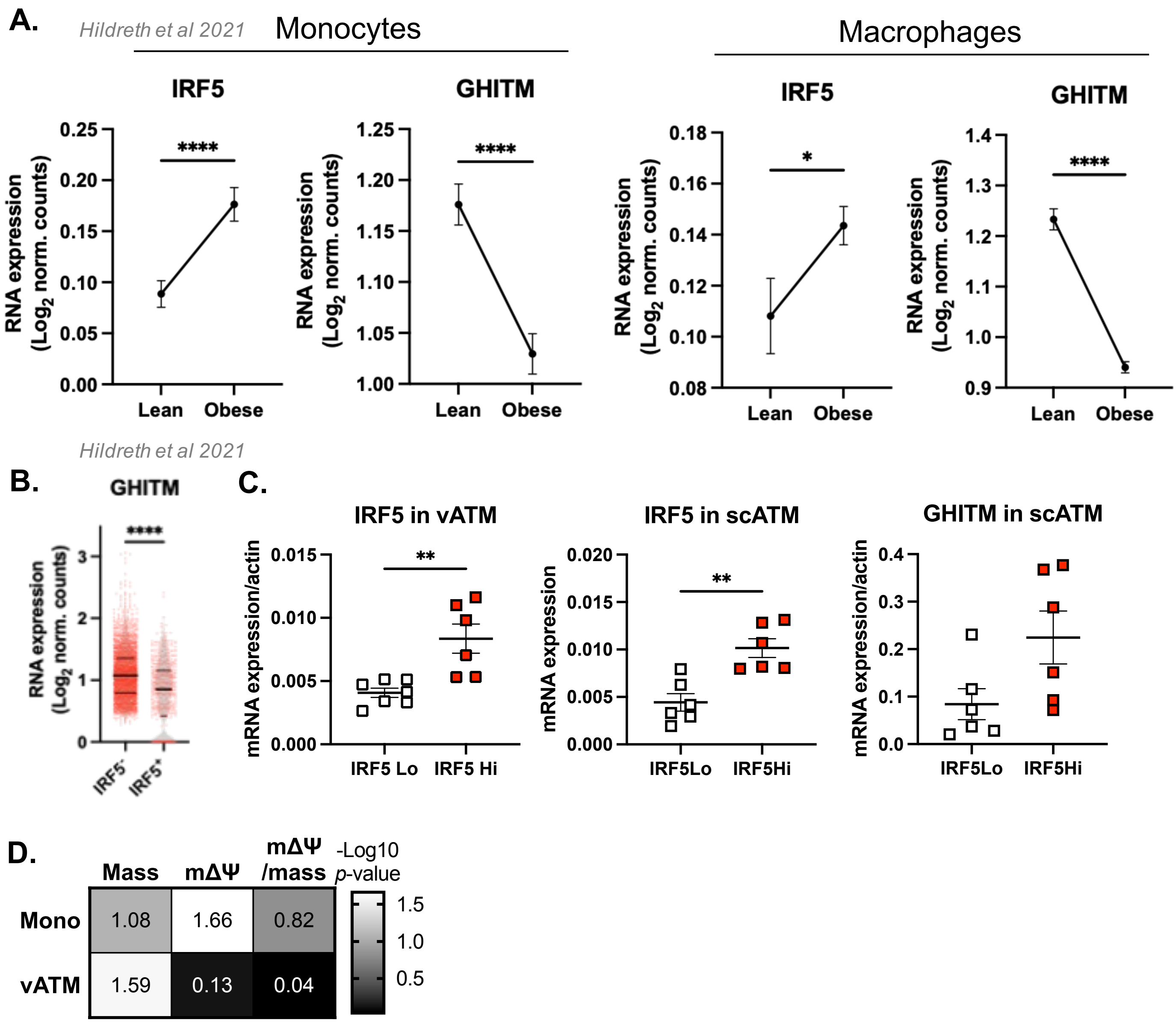

Figure S9. IRF5-GHITM coregulation is conserved in humans

A. IRF5 and GHITM RNA counts in visceral AT (WAT) macrophages (top) and monocytes (bottom), from single-cell RNA sequencing of the stromal vascular fraction of WAT from individuals with obesity or that are lean (Hildreth *et al* 2021) (n=532 monocytes sequenced from individual that is lean, n=505 monocytes sequenced from individual with obesity; n=498 macrophages sequenced from individual that is lean and n=1393 macrophages sequenced from individual with obesity; left-to-right, two-tailed unpaired t-test ****p=0.000028, **** p=0.00000026, *p=0.021, ****$p<1.0x10^{-15}$).

B. GHITM RNA expression in $IRF5^{-}$ and $IRF5^{+}$ cells (Hildreth *et al* 2021) (n=2959 cells sequenced and split into $IRF5^{-}$, n=2411, or $IRF5^{+}$, n=548, based on detectable expression of *Irf5*. Two-tailed unpaired t-test **** $p<1.0x10^{-15}$).

C. RNA expression of IRF5 normalized to actin in $CD14^{+}$ human visceral (vATMs) and subcutaneous adipose tissue macrophages (scATMs). GHITM RNA expression normalised to actin in $CD14^{+}$ scATMs. Samples were stratified based on their expression level of *Irf5* into IRF5 Lo versus IRF5 Hi expressors (n=7 for IRF5 Lo and n=6 for IRF5 Hi).

D. Correlative analyses of MFI of IRF5, JC1-Green (mitochondrial mass, Mt Mass), JC1-Red (mΔΨ), and JC1-Red-to-Green ratio (mΔΨ/mass) in human visceral ATMs (vATM) from patients with obesity and in monocytes from patients with T2D (Mono) analysed by FACS (n=11 for monocytes, n=9 for ATMs, Pearson's correlation, $-Log^{10}$ of two-tailed p-value shown, Pearson r in Fig 7H).

Data are presented as mean ± SEM. Source data are provided as a Source Data file.

Supplementary Tables

Table S1. Genotyping primers

Genotyping		
**IRF5 WT**	F	CGT GTA GCA CTC CAT GCT CT
	R	AGG GCC TGT CCA GAA TTAGG
**IRF5 Mut**	F	CTT CGT ATA GCA TAC ATT ATACG
	R	AGG GCC TGT CCA GAA TTAGG
**LyzM WT**	F	TTA CAG TCG GCC AGG CTG AC
	R	CTT GGG CTG CCA GAA TTTCTC
**LyzM Mut**	F	CCC AGA AAT GCC AGA TTA CG
	R	CTT GGG CTG CCA GAA TTTCTC
**Cas9 cre std WT**	F	AAGGGAGCTGCAGTGGAGTA
	R	CAGGACAACGCCCACACA
**Cas9 cre std MUT**	F	CTTCTTCTTTGGGGCCATCT
	R	TCCCCATCAAGCTGATCC

Table S2. Surface markers for FACS analyses

Antibody	Dye	Clone	Reference
**Human**			
**HLA-DR**	Vioblue	AC122	Miltenyi ; 130-095-293
**CD19**	BV510	HIB19	BioLegend ; 302242
**CD14**	PE-Cy7	M5E2	BioLegend ; 301814
**CD16**	APC	3G8	BioLegend ; 302012
**CD45**	APC-eFluor780	HI30	eBioscience ; 47-0459-42
**CD31**	BV510	M89D3	BD ; 744463
**CD11c**	PerCP-Cy5.5	Bu15	Biolegend ; 337209
**CD206**	APC	15-2	BioLegend ; 321110
**Mouse**			
**Antibody**	Dye	Clone	Reference
**CD206**	BV421	C068C2	Biolegend ; 141717
**CD19**	BV510	6D5	Biolegend ; 115546
**CD3**	BV510	17A2	Biolegend ; 100234
**CD45**	PE-eF610	30-F11	Invitrogen ; 61-0451-82
**F4/80**	PE-Cy7	BM8	Invitrogen ; 25-4801-82
**CD11c**	APC	N418	Invitrogen ; 17-0114-81
**CD11b**	APC-Cy7	M1/70	Biolegend ; 101226

Table S3. Sequence-specific primers for quantitative RT-PCR

**Murine primers**		
**Irf5**	F	GATGGGGACAACACCATCTT
	R	GGCTTTTGTTAAGGGCACAG
**TNF**	F	CCACCACGCTCTTCTGTCTA
	R	CACTTGGTGGTTTGCTACGA
**IL6**	F	TACCACTTCACAAGTCGGAGGC
	R	CTGCAAGTGCATCATCGTTGTTC
**GHITM**	F	CTGCATTCTGGTGTGATGGG
	R	TGAGTACAGAGTGGCACCAG
**F4/80**	F	CCTTGGGAGCCTTCTGGATC
	R	CATCTGTGGCTGCCTCCCT
**MCP1**	F	GGGCCTGCTGTTCACAGTT
	R	CCAGCCTACTCATTGGGAT
**18S**	F	GGGAGCCTGAGAAACGGC
	R	GGGTCGGGAGTGGGTAATTT
**Actin**	F	GGCTGTATTCCCCTCCATCG
	R	CCAGTTGGTAACAATGCCATGT
**CD206**	F	TGTGGTGAGCTGAAAGGTGA
	R	CAGGTGTGGGCTCAGGTAGT
**CD11c**	F	CCATTTGCTTCCTCCAACAT
	R	GAGAGCCCAGACGAAGACAG
**Human primers**		
**GHITM**	F	TTCCATCACGAAGAATCAATGGC
	R	CCATAGTAGCACAATGCTCCAA
**IRF5**	F	CTCTTGTTAAGGGCACAGC
	R	AACACCATCTTCAAGGCCT
**Actin**	F	GGACTTCGAGCAAGAGATGG
	R	AGCACTGTGTTGGCGTACAG

Supplementary Methods

Gating strategies

Analysis of murine adipose tissue macrophages (ATM). Fig. 1D, F, H, I, J, K, Fig S2A, C, D, E, F, Fig. S3B, C and D. Median fluorescence intensity (MFI) for IRF5 expression, BODIPY, JC1-Red and JC1-Green were exported from populations of (**A**) adipose tissue macrophages (ATM) and from (**B**) F4/80^{Hi} and F4/80^{Lo} ATMs.

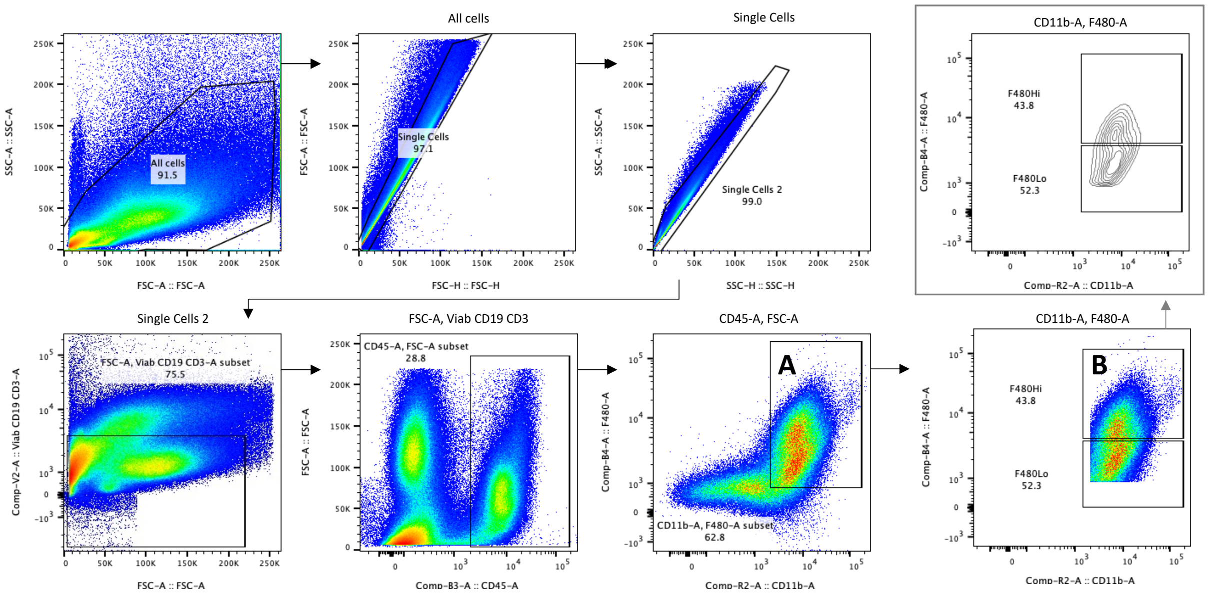

Analysis of human adipose tissue macrophages (ATM). Fig. 7H and Fig. S9D. MFI for IRF5 expression, JC1-Red and JC1-Green were exported from populations of (**A**) adipose tissue macrophages (ATM).

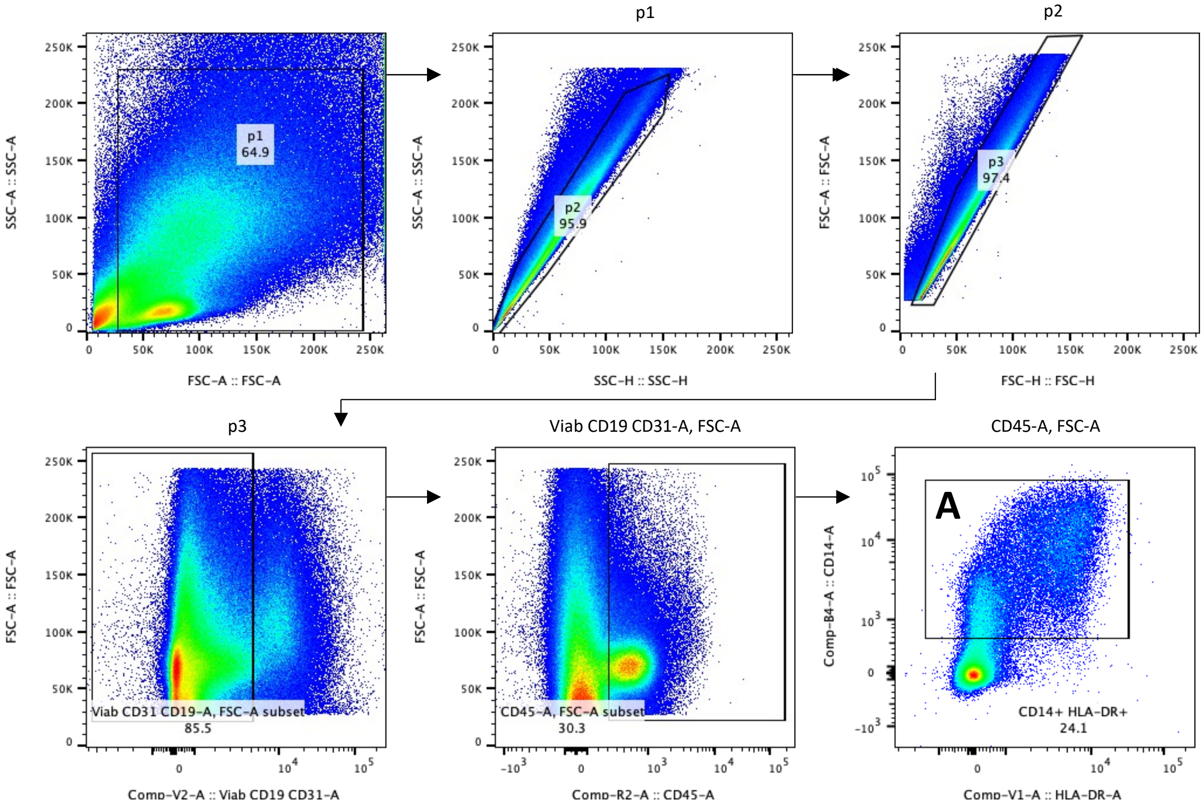

Analysis of human monocytes. Fig. 7H and Fig. S9D. Analysis of human monocytes. MFI for IRF5 expression, JC1-Red and JC1-Green were exported from populations of (**A**) monocytes.

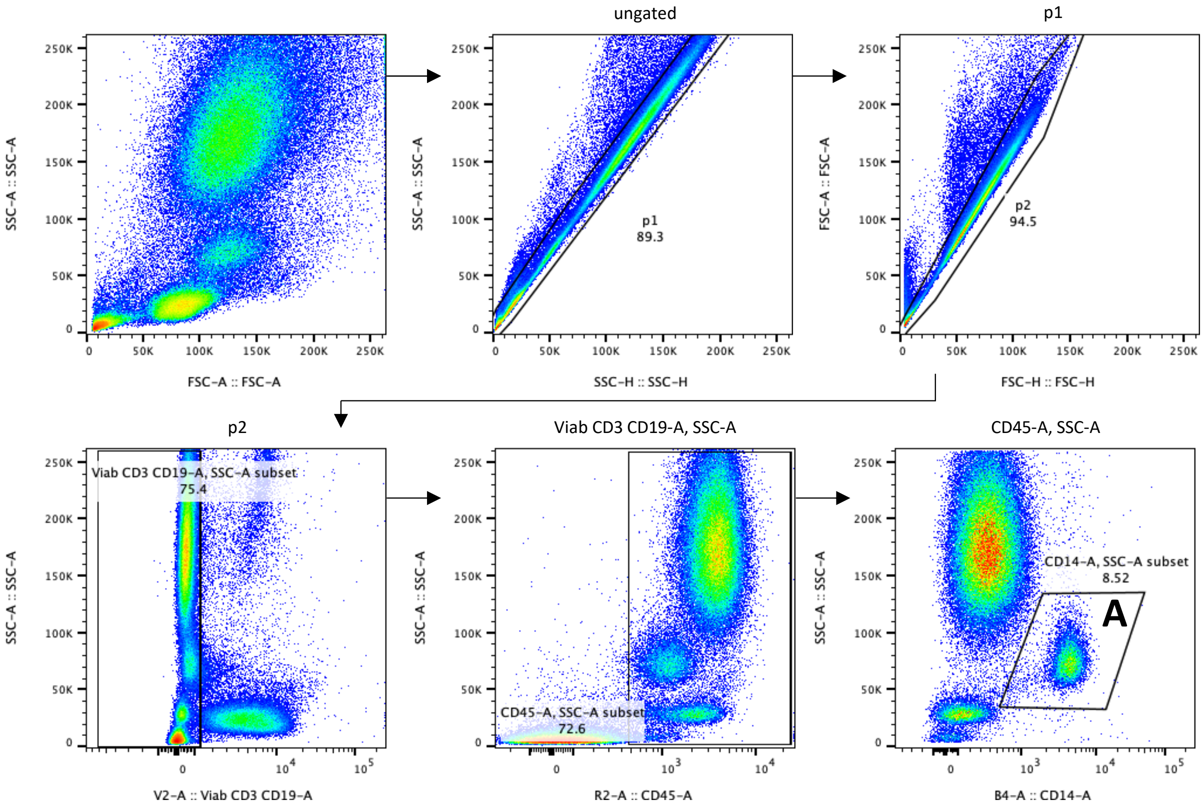

Sorting strategy of human monocytes. Fig. 7A. Lineage markers were used to exclude non-monocyte cells, monocytes where gated based on IRF5 expression and then gated for viability and doublet exclusion. IRF5+ (**A**) and IRF5- (**B**) monocytes were sorted.

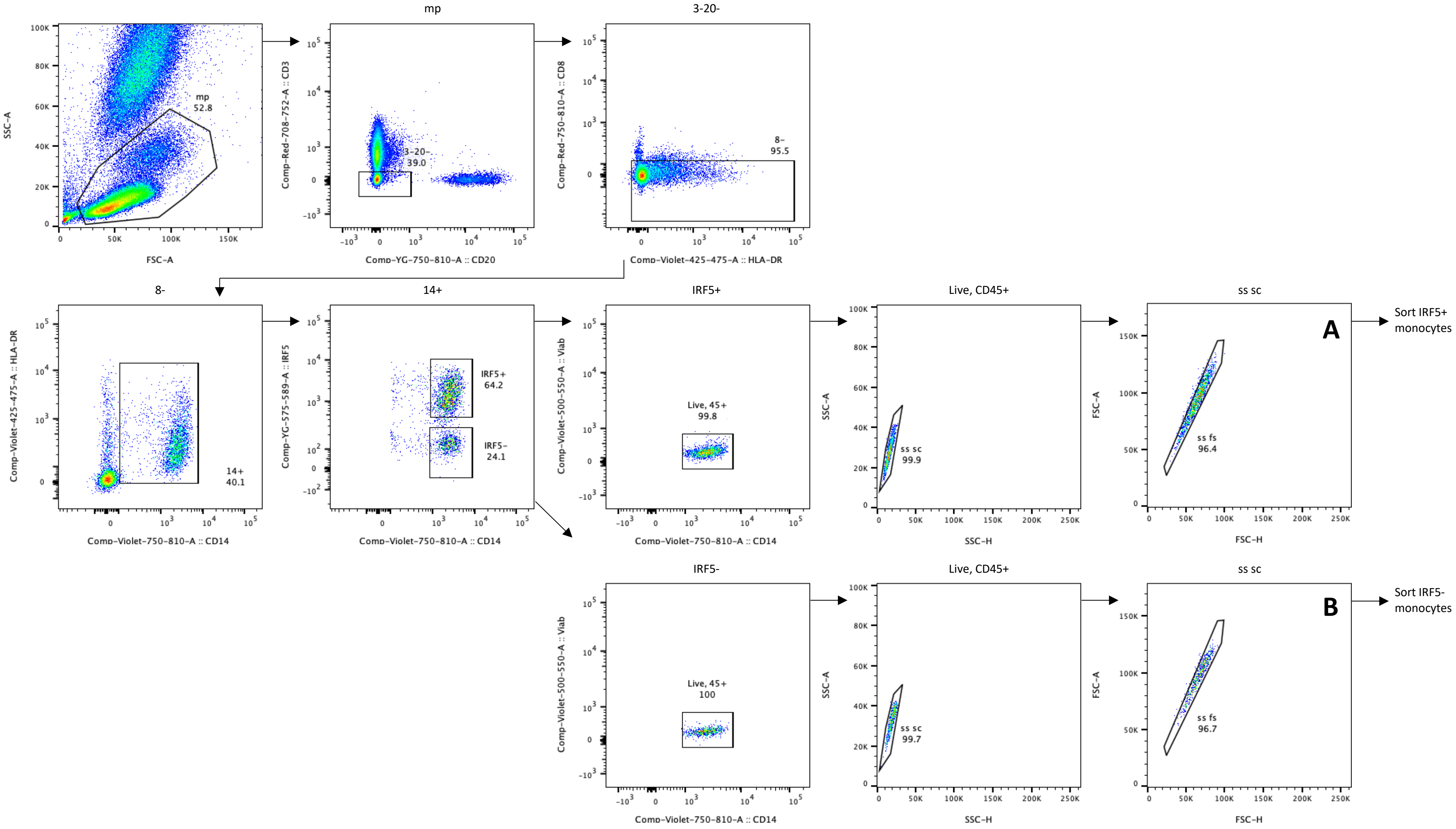

Supplement: Supplementary file 1 — Supplementary Information [file 41467_2022_32813_MOESM1_ESM.pdf]
