# Supplementary material for: Early macrophage response to obesity encompasses Interferon Regulatory Factor 5 regulated mitochondrial architecture remodelling
Source: Nat Commun. 2022 Aug 30;13:5089. doi: 10.1038/s41467-022-32813-z (PMC9427774; doi:10.1038/s41467-022-32813-z)
Supplement: Supplementary file 3 — Reporting Summary [file 41467_2022_32813_MOESM3_ESM.pdf]

nature research

Corresponding author(s): Fawaz Alzaid, Nicolas Venteclef

Last updated by author(s): Jul 23, 2022

Reporting Summary

Nature Research wishes to improve the reproducibility of the work that we publish. This form provides structure for consistency and transparency in reporting. For further information on Nature Research policies, see our Editorial Policies and the Editorial Policy Checklist.

Statistics

For all statistical analyses, confirm that the following items are present in the figure legend, table legend, main text, or Methods section.

n/a	Confirmed	
☐	☒	The exact sample size (*n*) for each experimental group/condition, given as a discrete number and unit of measurement
☐	☒	A statement on whether measurements were taken from distinct samples or whether the same sample was measured repeatedly
☐	☒	The statistical test(s) used AND whether they are one- or two-sided<br>*Only common tests should be described solely by name; describe more complex techniques in the Methods section.*
☒	☐	A description of all covariates tested
☒	☐	A description of any assumptions or corrections, such as tests of normality and adjustment for multiple comparisons
☐	☒	A full description of the statistical parameters including central tendency (e.g. means) or other basic estimates (e.g. regression coefficient) AND variation (e.g. standard deviation) or associated estimates of uncertainty (e.g. confidence intervals)
☐	☒	For null hypothesis testing, the test statistic (e.g. *F*, *t*, *r*) with confidence intervals, effect sizes, degrees of freedom and *P* value noted<br>*Give P values as exact values whenever suitable.*
☒	☐	For Bayesian analysis, information on the choice of priors and Markov chain Monte Carlo settings
☒	☐	For hierarchical and complex designs, identification of the appropriate level for tests and full reporting of outcomes
☐	☒	Estimates of effect sizes (e.g. Cohen's *d*, Pearson's *r*), indicating how they were calculated

Our web collection on statistics for biologists contains articles on many of the points above.

Software and code

Policy information about availability of computer code

Data collection	-BioTuring Browser software v2.7.48 (https://bioturing.com/) was used to access and analyse previously published single cell sequencing data by Jaitin et al 2019 (GSE128518: https://www.ncbi.nlm.nih.gov/geo/query/acc.cgi?acc=GSE128518).<br>-UCSC genome browser (https://genome.ucsc.edu) was used to access and visualize gene tracks A live link to session is provided.<br>-BD FACSDivaTM sotware v9.0 (BD) (https://www.bdbiosciences.com/en-ca/products/software/instrument-software/bd-facsdiva-software).<br>-MACSQuantifyTM v2.11 (Miltenyi Biotec) (https://www.miltenyibiotec.com/US-en/products/macsquantify-software.html#gref).<br>-BD FACSDivaTM and MACSQuantifyTM were used to collect flow cytometry data.<br>-Quant Studio 3 Operating software v1.5.1 and Design and Analysis software v1.5.2 (ThermoFisher Scientific) were used to collect gene expression data (https://www.thermofisher.com/kw/en/home/global/forms/life-science/quantstudio-3-5-software.html).<br>-Wave Desktop v16.5 (Agilent) was used to collect metabolic flux analysis data (https://www.agilent.com/en/products/cell-analysis/software-download-for-wave-desktop).<br>-Xcalibur v2.1 (ThermoFisher Scientific) was used to collect data from LC-HRMS experiments.<br>-2100 Expert Software vB.01.11(SR1) (Agilent) (https://explore.agilent.com/Software-Download-2100-Expert?productURL=https%3A%2F%2Fwww.agilent.com%2Fen%2Fproduct%2Fautomated-electrophoresis%2Fbioanalyzer-systems%2Fbioanalyzer-software%2F2100-expert-software-228259).<br>-Fusion Pulase TS v17.01 (Vilber) software was used to collect gel and membrane images.
Data analysis	STAR v2.7.3a and v2.5.2b (https://github.com/alexdobin/STAR)<br>DESeq2 v1.8.1 (http://bioconductor.org/packages/release/bioc/vignettes/DESeq2/inst/doc/DESeq2.html)<br>clusterProfiler v3.10.1 (https://bioconductor.org/packages/release/bioc/vignettes/clusterProfiler/inst/doc/clusterProfiler.html)<br>Panther Classification System v17.0 (http://www.pantherdb.org/).<br>PacBio workflow codes v1.0.0 are available at: https://github.com/LiLabZhaohua/PacBioWorkflow.<br>Orange3 software Version 3.28.0 (https://orangedatamining.com/) was used to create heatmaps for RNAseq data<br>FlowJo v10.8.1 (BD) to analyze FACS data (https://www.flowjo.com/solutions/flowjo/downloads)

Graphpad Prism v9.4.0 (GraphPad Software LLC) (https://www.graphpad.com/scientific-software/prism/)
Fiji (ImageJ2) v2.3.0/1.53q (https://imagej.net/software/fiji/downloads)

For manuscripts utilizing custom algorithms or software that are central to the research but not yet described in published literature, software must be made available to editors and reviewers. We strongly encourage code deposition in a community repository (e.g. GitHub). See the Nature Research guidelines for submitting code & software for further information.

Data

Policy information about availability of data

All manuscripts must include a data availability statement. This statement should provide the following information, where applicable:

- Accession codes, unique identifiers, or web links for publicly available datasets
- A list of figures that have associated raw data
- A description of any restrictions on data availability

The following data availability statement is provided in the manuscript:

Data availability: Gene raw counts and raw fastq files for RNA-seq data generated in this study are available on GEO repository (www.ncbi.nlm.nih.gov/geo/). RNA-seq of IRF5+/- human monocytes (Fig. 7A, B, C) available under accession number: GSE176216 (https://www.ncbi.nlm.nih.gov/geo/query/acc.cgi?acc=GSE176216; GSM5360191-4 and GSM5360167-70 not included in study). RNA-seq of F4/80+ ATMs and BMDM from IRF5-KO and WT mice (Fig. 1A, B; Fig. 5A, B, D, E, Fig. S1A, B; Fig. S7A) available under accession numbers: GSE208648 and GSE208667, respectively. (https://www.ncbi.nlm.nih.gov/geo/query/acc.cgi?acc=GSE208648 and https://www.ncbi.nlm.nih.gov/geo/query/acc.cgi?acc=GSE208667). Previously published dataset analysed in this paper are from Jaitin et al 201912 (Fig. 6A, B, C, D; Fig. S8A) (GSE128518, https://www.ncbi.nlm.nih.gov/geo/query/acc.cgi?acc=GSE128518) and from Hildreth et al 202132 (Fig. 7D, E, F; Fig. S9A, B) (GSE155960, https://www.ncbi.nlm.nih.gov/geo/query/acc.cgi?acc=GSE155960). ChIP-seq data from Saliba et al 201431 (Fig. 5C, D; Fig. S7B) is available under accession number E-MTAB-2661 (https://www.ebi.ac.uk/arrayexpress/experiments/E-MTAB-2661/). The GRCh38/hg38 assembly used is accessible via GenBank/RefSeq assembly accession numbers GCA_000001405.15/GCA_000001405.26 (https://www.ncbi.nlm.nih.gov/assembly/GCF_000001405.26).
Source data are provided with this paper.

Field-specific reporting

Please select the one below that is the best fit for your research. If you are not sure, read the appropriate sections before making your selection.

- [x] Life sciences
- [ ] Behavioural & social sciences
- [ ] Ecological, evolutionary & environmental sciences

For a reference copy of the document with all sections, see nature.com/documents/nr-reporting-summary-flat.pdf

Life sciences study design

All studies must disclose on these points even when the disclosure is negative.

Sample size	Sample sizes were based on our previous experience with models (Dalmas et al 2015 Nat Med doi: 10.1038/nm.3829; Alzaid et al 2016 JCI Insight doi: 10.1172/jci.insight.88689; Alzaid et al 2020 EMBO Mol Med doi: 10.15252/emmm.202013038).
Data exclusions	No data were excluded from the study
Replication	All in vivo and in vitro experiments were replicated in 3 independent experiments, all replications were successful. Experiments with human samples were not replicated in independent cohorts. One independent experimental cohort was used to generate each set of data (Fig. 7A-C, Fig. 7G, Fig. 7H, Fig. 7I). Each set included biological replicates (biologically independent n are cited in figure legends) and technical replicates were included for sequencing (cited in methods). All replications were successful.
Randomization	Allocation to groups was based on genotype for mice and primary culture. Allocation to groups in humans is described in figure legends, samples were allocated to groups based on biological grouping variables (expression of marker gene or protein).
Blinding	Investigators were blinded during analyses for sequencing. Investigators were blinded for data collection and analyses for electron microscopy on IRF5-KO and WT-derived samples. Investigators were blinded during data collection for in vivo metabolic testing on IRF5-KO and WT mice. Investigators were not blinded for in vivo metabolic testing on ST-HFD and LT-HFD models applied to C57BL/6J mice, blinding was not possible as experimental groups had visibly different weight gain. For downstream experimentation on samples derived from in vivo experiments (FACS, gene expression, metabolic flux analyses, histology) the investigator organizing the experimental groups and involved in sample collection was not blinded; colleagues aiding in data collection were blinded. For in vitro experiments, the investigators were not blinded for group allocation as the same investigator both planned and performed the experiment.

Reporting for specific materials, systems and methods

We require information from authors about some types of materials, experimental systems and methods used in many studies. Here, indicate whether each material, system or method listed is relevant to your study. If you are not sure if a list item applies to your research, read the appropriate section before selecting a response.

Materials & experimental systems

n/a	Involved in the study
☐	☒ Antibodies
☒	☐ Eukaryotic cell lines
☒	☐ Palaeontology and archaeology
☐	☒ Animals and other organisms
☐	☒ Human research participants
☒	☐ Clinical data
☒	☐ Dual use research of concern

Methods

n/a	Involved in the study
☒	☐ ChIP-seq
☐	☒ Flow cytometry
☒	☐ MRI-based neuroimaging

Antibodies

Antibodies used

Antibodies are listed in "Supplementary Table S2. List of antibodies" and in methods section. These are:
Abcam, Actin, Cat#ab8226, dilution 1:1000
Abcam, Oxphos cocktail, Cat#MS604, dilution 1:100
BD, CD31 BV510, Cat#744463, clone M89D3, dilution 1:200, lot#9182228
BioLegend, CD11b APC-Cy7, Cat#101226, clone M1/70, dilution 1:300, lot#B259668
BioLegend, CD11c PerCP-Cy5.5, Cat#337209, clone Bu15, dilution 1:200, lot#B262407
BioLegend, CD14 PE-Cy7, Cat#301814, clone M5E2, dilution 1:200, lot#B272337
BioLegend, CD16 APC, Cat#302012, clone 3G8, dilution 1:200, lot#B263227
BioLegend, CD19 BV510, Cat#115546, clone 6D5, dilution 1:200, lot#B255308
BioLegend, CD19 BV510, Cat#302242, clone HIB19, dilution 1:200, lot#B242298
BioLegend, CD206 APC, Cat#321110, clone 15-2, dilution 1:200, lot#B265383
BioLegend, CD206 BV421, Cat#141717, clone C068C2, dilution 1:50, lot#B342526
BioLegend, CD3 BV510, Cat#100234, clone 17A2, dilution 1:50, lot#B343121
eBioscience, CD45 APC-eFluor780, Cat#47-0459-42, clone HI30, dilution 1:200, lot#E11369-1636
Invitrogen, anti-mouse FITC, Cat#A11001, dilution 1:200
Invitrogen, anti-mouse HRP, Cat#31430, dilution 1:1000
Invitrogen, anti-rabbit AF555, Cat#A21428, dilution 1:200
Invitrogen, anti-rabbit HRP, Cat#31460, dilution 1:1000
Invitrogen, CD11c APC, Cat#17-0114-81, clone N418, dilution 1:100, lot#2271166
Invitrogen, CD14, Cat#13-0149-82, dilution 1:100
Invitrogen, CD45 PE-eF610, Cat#61-0451-82, clone 30-F11, dilution 1:200, lot#2006524
Invitrogen, F4/80 PE-Cy7, Cat#25-4801-82, clone BM8, dilution 1:200, lot#4323732
Miltenyi, HLA-DR Vioblue, Cat#130-095-293, clone AC122, dilution 1:200, lot#5200609083
Proteintech, GHITM, Cat#16296-1-AP, dilution 1:500
Proteintech, IRF5, Cat#10547-1-AP, dilution 1:200 for FACS, 1:30 for imaging
ThermoFisher Scientific, anti-rabbit PE, Cat#12-4739-81, dilution 1:50
ThermoFisher Scientific, streptavidin AF647, Cat#S32357, dilution 1:200

Validation

Abcam, Actin, Cat#ab8226, dilution 1:1000, QC testing, reactivity mouse, application western blot (https://www.abcam.com/beta-actin-antibody-mabcam-8226-loading-control-ab8226.html).
Abcam, Oxphos cocktail, Cat#MS604, dilution 1:100, QC testing, reactivity mouse/human, commercialised cocktail application immunoblot (individual antibodies validated for imaging ab110242, ab14705, ab14714, ab14745, ab14748) (https://www.abcam.com/total-oxphos-rodent-wb-antibody-cocktail-ab110413.html).
BD, CD31 BV510, Cat#744463, clone M89D3, dilution 1:200, lot#9182228, QC testing, reactivity human, application flow cytometry (https://www.bdbiosciences.com/en-eu/products/reagents/flow-cytometry-reagents/research-reagents/single-color-antibodies-ruo/bv510-mouse-anti-human-cd31.744463).
BioLegend, CD11b APC-Cy7, Cat#101226, clone M1/70, dilution 1:300, lot#B259668, QC testing, reactivity mouse/human, application flow cytometry (https://www.biolegend.com/fr-fr/products/apc-cyanine7-anti-mouse-human-cd11b-antibody-3930).
BioLegend, CD11c PerCP-Cy5.5, Cat#337209, clone Bu15, dilution 1:200, lot#B262407, QC testing, reactivity human, application flow cytometry (https://www.biolegend.com/fr-fr/products/percp-cyanine5-5-anti-human-cd11c-antibody-5397).
BioLegend, CD14 PE-Cy7, Cat#301814, clone M5E2, dilution 1:200, lot#B272337, QC testing, reactivity human, application flow cytometry (https://www.biolegend.com/en-gb/products/pe-cyanine7-anti-human-cd14-antibody-2729).
BioLegend, CD16 APC, Cat#302012, clone 3G8, dilution 1:200, lot#B263227, QC testing, reactivity human, application flow cytometry (https://www.biolegend.com/en-gb/products/apc-anti-human-cd16-antibody-565).
BioLegend, CD19 BV510, Cat#115546, clone 6D5, dilution 1:200, lot#B255308, QC testing, reactivity mouse, application flow cytometry (https://www.biolegend.com/fr-fr/products/brilliant-violet-510-anti-mouse-cd19-antibody-8563).
BioLegend, CD19 BV510, Cat#302242, clone HIB19, dilution 1:200, lot#B242298, QC testing, reactivity human, application flow cytometry (https://www.biolegend.com/en-gb/products/brilliant-violet-510-anti-human-cd19-antibody-8004).
BioLegend, CD206 APC, Cat#321110, clone 15-2, dilution 1:200, lot#B265383, QC testing, reactivity human, application flow cytometry (https://www.biolegend.com/fr-fr/products/apc-anti-human-cd206-mmr-antibody-2996).
BioLegend, CD206 BV421, Cat#141717, clone C068C2, dilution 1:50, lot#B342526, QC testing, reactivity mouse, application flow cytometry (https://www.biolegend.com/fr-fr/products/brilliant-violet-421-anti-mouse-cd206-mmr-antibody-8638).
BioLegend, CD3 BV510, Cat#100234, clone 17A2, dilution 1:50, lot#B343121, QC testing, reactivity mouse, application flow cytometry (https://www.biolegend.com/fr-fr/products/brilliant-violet-510-anti-mouse-cd3-antibody-7990).
eBioscience, CD45 APC-eFluor780, Cat#47-0459-42, clone HI30, dilution 1:200, lot#E11369-1636, QC testing, reactivity human, application flow cytometry (https://www.thermofisher.com/antibody/product/CD45-Antibody-clone-HI30-Monoclonal/47-0459-42).
Invitrogen, anti-mouse FITC, Cat#A11001, dilution 1:200, QC testing, reactivity mouse, application imaging (https://www.thermofisher.com/antibody/product/Goat-anti-Mouse-IgG-H-L-Cross-Adsorbed-Secondary-Antibody-Polyclonal/A-11001).

Invitrogen, anti-mouse HRP, Cat#31430, dilution 1:1000, QC testing, reactivity mouse, application western blot (https://www.thermofisher.com/antibody/product/Goat-anti-Mouse-IgG-H-L-Secondary-Antibody-Polyclonal/31430).
Invitrogen, anti-rabbit AF555, Cat#A21428, dilution 1:200, QC testing, reactivity rabbit, application imaging (https://www.thermofisher.com/antibody/product/Goat-anti-Rabbit-IgG-H-L-Cross-Adsorbed-Secondary-Antibody-Polyclonal/A-21428).
Invitrogen, anti-rabbit HRP, Cat#31460, dilution 1:1000, QC testing, reactivity mouse, application western blot (https://www.thermofisher.com/antibody/product/Goat-anti-Rabbit-IgG-H-L-Secondary-Antibody-Polyclonal/31460).
Invitrogen, CD11c APC, Cat#17-0114-81, clone N418, dilution 1:100, lot#2271166, QC testing, reactivity mouse, application flow cytometry (https://www.thermofisher.com/antibody/product/CD11c-Antibody-clone-N418-Monoclonal/17-0114-82).
Invitrogen, CD14, Cat#13-0149-82, dilution 1:100, QC testing, reactivity human, application flow cytometry (supplier), imaging (published reported by supplier) (https://www.thermofisher.com/antibody/product/CD14-Antibody-clone-61D3-Monoclonal/13-0149-82).
Invitrogen, CD45 PE-eF610, Cat#61-0451-82, clone 30-F11, dilution 1:200, lot#2006524, QC testing, reactivity mouse, application flow cytometry (https://www.thermofisher.com/antibody/product/CD45-Antibody-clone-30-F11-Monoclonal/61-0451-82).
Invitrogen, F4/80 PE-Cy7, Cat#25-4801-82, clone BM8, dilution 1:200, lot#4323732, QC testing, reactivity mouse, application flow cytometry (https://www.thermofisher.com/antibody/product/F4-80-Antibody-clone-BM8-Monoclonal/25-4801-82).
Miltenyi, HLA-DR Vioblue, Cat#130-095-293, clone AC122, dilution 1:200, lot#5200609083, QC testing, reactivity human, application flow cytometry (https://www.miltenyibiotec.com/US-en/products/hla-dr-antibody-anti-human-ac122.html#vioblue:30-tests-in-60-ul).
Proteintech, GHITM, Cat#16296-1-AP, dilution 1:500, QC testing, reactivity mouse/human, application western blot/imaging (https://www.ptglab.com/products/GHITM-Antibody-16296-1-AP.htm).
Proteintech, IRF5, Cat#10547-1-AP, dilution 1:200 for FACS, 1:30 for imaging, QC testing, reactivity mouse/human, application flow cytometry/imaging (https://www.ptglab.com/products/IRF5-Antibody-10547-1-AP.htm).
ThermoFisher Scientific, anti-rabbit PE, Cat#12-4739-81, dilution 1:50, QC testing, reactivity rabbit, application flow cytometry (https://www.thermofisher.com/antibody/product/Donkey-anti-Rabbit-IgG-H-L-Secondary-Antibody-Polyclonal/12-4739-81).
ThermoFisher Scientific, streptavidin AF647, Cat#S32357, dilution 1:200, QC testing, reactivity human, application flow cytometry/imaging (https://www.thermofisher.com/order/catalog/product/S32357?SID=srch-srp-S32357).

Animals and other organisms

Policy information about studies involving animals; ARRIVE guidelines recommended for reporting animal research

Laboratory animals

Male C57BL/6J mice (5-7 weeks) were purchased from Charles River. To generate mice with a myeloid-specific deletion of IRF5, IRF5 flox/flox mice (C57BL/6-Irf5tm1Ppr/J; stock no. 017311) were crossed with LysM-Cre mice (B6.129P2-Lyz2tm1(cre)Ifo/J; stock no. 04781), purchased from The Jackson Laboratory. To generate mice with a restricted myeloid expression of the Cas9 endonuclease, Rosa26-Cas9KI mice (Gt(ROSA)26Sortm1.1(CAG-cas9*,-EGFP)Fezh/J; stock no. 024858, The Jackson Laboratory) were crossed with LysM-Cre mice. Mice were housed at 22°C and 50% humidity, on average, on a 12 h light/dark cycle in the “Centre d’Explorations Fonctionnelles” of Sorbonne University (UMS-28). All mice used in the study were male and aged between 7-10 weeks old at the time of the experiment starting point. Number of mice used per experiment is detailed in figure legends.

Wild animals

The study did not involve wild animals

Field-collected samples

The study did not involve field collected samples

Ethics oversight

All animal experiments were approved by the French ethical board (Paris-Sorbonne University, Charles Darwin N°5, 01026.02; protocols #11545, #11546, #22537, #17001), experiments were conducted in accordance with the guidelines stated in the International Guiding Principles for Biomedical Research Involving Animals.

Note that full information on the approval of the study protocol must also be provided in the manuscript.

Human research participants

Policy information about studies involving human research participants

Population characteristics

Patients were consecutively recruited and samples obtained from patients with type-2 diabetes or from obese patients as stated in Methods section.

Population characteristics in methods: Sorted and sequenced monocytes (Fig. 7A-C) were from patients with T2D aged 67-73 years old (4 male/1 female). Sorted ATMs (Fig. 7G) were from patients with obesity aged 37-54 years old (gender was anonymized for these patients). Samples analyzed by cytometry (Fig. 7H) were monocytes from patients with T2D aged 45-74 years old (6 male/ 7 female) and ATMs from patients with obesity aged 41-59 years old (1 male/8 female). Blood samples prepared for immunofluorescence (Fig. 7I) were from patients with T2D aged 47-81 years old (9 male/1 female).

Recruitment

No self-selection or group allocation biases impact the results to our knowledge. Group allocation was empirically determined based on sample intrinsic biological variables (e.g. IRF5+ versus IRF5- monocytes from the same patients, grouping as high versus low expressors of IRF5 in adipose tissue macrophages, nuclear versus cytoplasmic localization of IRF5).

Methods statement: Participants were consecutively recruited, blood samples and adipose tissue biopsies were obtained from different populations admitted to the Lariboisière and Geoffroy Saint Hilaire hospitals (Paris, France), respectively. Adipose tissue biopsies were obtained from obese subjects during bariatric surgery.

Ethics oversight

Methods statement: For work with human samples, the Ethics Committee of CPP Ile-de-France approved the clinical investigations for all individuals, and written informed consent was obtained from all individuals. The clinical trial principal

investigator is Prof. Jean-François Gautier: jean-francois.gautier@aphp.fr. Studies were conducted in accordance with the Helsinki Declaration and were registered to a public trial registry (Clinicaltrials.gov; NCT02671864).

Note that full information on the approval of the study protocol must also be provided in the manuscript.

Flow Cytometry

Plots

Confirm that:

☒ The axis labels state the marker and fluorochrome used (e.g. CD4-FITC).

☒ The axis scales are clearly visible. Include numbers along axes only for bottom left plot of group (a 'group' is an analysis of identical markers).

☒ All plots are contour plots with outliers or pseudocolor plots.

☒ A numerical value for number of cells or percentage (with statistics) is provided.

Methodology

Sample preparation	Cells were obtained as described in the methods section from blood or from the stromal vascular fraction of adipose tissue following disruption with collagenase. cells were resuspended in FACS buffer as described in the methods section and incubated with an Fc-blocker (120-000-422, Miltenyi Biotech) for 10 min. For metabolic analysis, cells were incubated with either 200 µM JC-1 (T3168, ThermoFisher Scientific) or 14.6 µM 2-NBDG (N13195, ThermoFisher Scientific) for 30 min at 37° C. Finally, cells were stained for surface markers (Table S2) and a Live/Dead viability dye (L34957, ThermoFisher Scientific) according to manufacturer's protocol. For IRF5 staining, cells were fixed with Foxp3-staining kit (00-5523-00, ThermoFisher Scientific) and then stained with an anti-IRF5 (10547-1-AP, Proteintech) for 1 h, and then with a secondary PE antibody (12-4739- 81, ThermoFisher Scientific) for 30 min. Acquisition was performed on a MACSQuant cytometer (Miltenyi Biotech). Cell sorting was performed on a FACSAria III (BD Biosciences). A list of antibodies and fluorophores is provided.
Instrument	Acquisition was performed on a MACSQuant cytometer (Miltenyi Biotech). Cell sorting was performed on a FACSAria III (BD Biosciences)
Software	BD FACSDivaTM and MACSQuantifyTM were used to collect flow cytometry data.<br>FlowJo v10.8.1 (BD) to analyze FACS data (https://www.flowjo.com/solutions/flowjo/downloads)
Cell population abundance	Cells were first test sorted into PBS and re-run through the cell sorter to ensure no contamination. Sorting was then continued directly in lysis buffer. Only sorting runs with more than 90% efficiency during the sort were used for downstream analyses.
Gating strategy	Full gating strategies are provided in supplementary methods.

☒ Tick this box to confirm that a figure exemplifying the gating strategy is provided in the Supplementary Information.
